# Safety of the COVID-19 vaccination in children with juvenile idiopathic arthritis—A observational study from two pediatric rheumatology centres in Poland

**DOI:** 10.3389/fped.2023.1103763

**Published:** 2023-03-10

**Authors:** Violetta Opoka-Winiarska, Joanna Lipinska, Arkadiusz Michalak, Jacek Burzyński, Olga Kądziołka, Elżbieta Smolewska

**Affiliations:** ^1^ Department of Paediatric Pulmonology and Rheumatology, Medical University of Lublin, Lublin, Poland; ^2^ Department of Paediatric Cardiology and Rheumatology, Medical University of Lodz, Lublin, Poland; ^3^ Department of Paediatrics, Diabetology, Endocrinology and Nephrology, Lodz, Poland; ^4^ Department of Biostatistics and Translational Medicine, Medical University of Lodz, Lodz, Poland; ^5^ Department of Paediatric Pulmonology and Rheumatology, University Children's Hospital of Lublin, Lublin, Poland

**Keywords:** juvenile idiopathic arthritis, COVID-19 vaccination, COVID-19, DMARDs, vaccine adverse reactions

## Abstract

**Introduction:**

The safety of COVID-19 vaccines in children with juvenile idiopathic arthritis (JIA) is the concern of patients and their parents and doctors in the current pandemic reality. The main objective of the study was to evaluate the safety of COVID-19 vaccine in patients with JIA.

**Method:**

A cohort study based on short clinical follow-up of 43 children with JIA was conducted in the years 2021–2022 in two centres of paediatric rheumatology in Poland. All patients received mRNA COVID-19 vaccine. The patients' data were collected using appropriate validated questionnaire. Disease activity was evaluated using Juvenile Arthritis Disease Activity Score 27-joint count (JADAS-27).

**Results:**

Ten (22.7%) children had COVID-19 infection before getting COVID-19 vaccine. After first dose of COVID-19 vaccine 25/43 (58.1%) patients presented typical adverse events: arm pain or oedema at the application side or weakness. Also, twenty five (58.1%) children had side effects after second dose of this vaccine, however the spectrum of the symptoms was wider (additionally: headache, fever, lymphadenopathy, arrhythmia). Thirteen out of 43 (30.2%) patients had active disease before and 8/43 (18.6%) after COVID-19 vaccination, while the degree of JADAS-27 activity was higher in the study group before COVID-19 vaccination (*p* = 0.047).

**Conclusions:**

Our study found out that children and adolescents with JIA with remission without treatment or on the long-term treatment—cDMARDs or even bDMARDs, can be safely vaccinated for COVID-19. Moreover, the study found that COVID-19 vaccination does not interfere with the JIA treatment and does not exacerbate symptoms of the disease and that vaccination protected against developing COVID-19 in children with JIA even on treatment.

## Key-points

Children with JIA with remission without treatment or on the long-term treatment conventional or even biological disease-modifying antirheumatic drugs (DMARDs), may be safely vaccinated for COVID-19.

COVID-19 vaccination does not interfere with the JIA treatment and does not exacerbate the rheumatoid process.

## Introduction

Vaccination is the most effective strategy to prevent and reduce the effects of the coronavirus disease 2019 (COVID-19) pandemic. Both randomized studies and further observations proved COVID-19 vaccines efficacy in reducing SARS-CoV-2 infection rates and severity of disease (1). However, patients with an immune dysfunction related either to the rheumatic disease or the use of immune-modulating drugs, could have altered COVID-19 vaccination response. Therefore, the answer to the question whether vaccination of a child with rheumatic disease is effective and safe is very important.

Juvenile idiopathic arthritis (JIA) is the most common rheumatic disease in childhood and includes various forms of arthritis, which begin before the age of 16 years, persist for more than 6 weeks and do not have determined cause. Therapeutic process involves treatment with disease-modifying antirheumatic drugs (DMARDs), including conventional (cDMARDs) and biological (bDMARDs) agents. In some cases, administration of short-term non-steroidal anti-inflammatory drugs and systemic or intraarticular glucocorticosteroids may be necessary (2).

Although adult and pediatric patients with rheumatic diseases treated with DMARDs do not seem to present a greater risk of COVID-19 or a worse disease outcome compared to the general population (3–5), some reports indicate a possible more severe course of the disease (6, 7).

The course of SARS-CoV-2 infection in patients with JIA is similar to the course of COVID-19 in the general pediatric population, despite JIA patients receiving immunosuppressive therapy. However, observations indicate that SARS-COV-2 infection may cause exacerbation of the disease, which required escalation of therapy (3, 7).

Vaccination of children with rheumatic disease is still important issue for rheumatologists and paediatricians. The Paediatric Rheumatology European Society (PRES), scientific society for healthcare professionals in paediatric rheumatology, has issued recommendations on vaccination for children with rheumatic diseases (8, 9). Despite the recommendation many children with JIA do not receive the COVID-19 vaccination due to fear of vaccine adverse events. In our experience parents and patients, as well as, health professionals are concerned about the exacerbation of the underlying disease and the unknown effects of the new vaccine. Faced with many questions and concerns from parents, patients, as well as, doctors about COVID-19 vaccination in children with rheumatic diseases, the aim of our study was an assessment of the COVID-19 vaccine safety and effectiveness in children and adolescents with JIA. The main objective of the study was to evaluate the incidence of adverse events after COVID-19 vaccine in patients with JIA and the impact of vaccination on disease activity.

## Materials and methods

### Study design

A cohort study based on a 6-week clinical follow-up of children with JIA was conducted in the years 2021–2022 in two centres of paediatric rheumatology in Poland: Department of Pediatric Pneumonology and Rheumatology, Medical University of Lublin and Department of Pediatric Cardiology and Rheumatology, Medical University of Lodz for a regular check-up visit.

### Study population

The inclusion criteria to the study were the diagnosis of JIA according to the 2001 Edmonton ILAR classification criteria (10), receiving two doses of COVID-19 vaccine and at least 6 weeks of follow-up from 2nd dose with no significant change in therapy. All patients received Pfizer-BioNTech mRNA COVID-19 vaccine (Comirnaty) in recommended doses (9). All guardians / parents and patients (obligatory aged 16 and over) agreed to participate in this observational study and signed the informed consent.

### Ethical approval

The study was approved by the Bioethics Committee at the Medical University of Lublin: KE-0254/209/2021. This study was conducted according to the principles of the Declaration of Helsinki and patient consent was obtained.

### Demographic, clinical and questionnaire data

Patients were evaluated at three time points: before vaccination (point 0), 6 weeks and 3 months if available after second dose of COVID-19 vaccination. Disease activity was assessed before and 6 weeks after getting COVID-19 vaccine.

To evaluate the safety of the vaccine, we analysed vaccination adverse events after the first and second COVID-19 vaccination doses and disease activity before and after both vaccination doses.

To assess the effectiveness of the vaccination, we followed the incidence of COVID-19 for 3 months after COVID-19 vaccination.

The patient's data regarding COVID-19 infection and COVID-19 vaccination including vaccine adverse events record was collected using questionnaire. The information concerned the JIA subtype, disease activity, JIA treatment was completed by the attending rheumatologist. In addition, the questionnaire included questions about the patient history of mandatory and recommended immunization in accordance with the Polish immunization program and COVID-19 vaccination in his family and siblings.

Disease activity was assessed on the basis of an interview, physical examination and the results of additional laboratory tests. Disease activity was evaluated by the patient and guardian/parent (parent/patient global assessment, PGA) and by rheumatologist (Physician global assessment, PhGA), using Visual Activity Scale (VAS) (1–10 cm), 0—means inactive disease and 10—maximum activity.

Laboratory inflammation markers: C-reactive protein (CRP) (cut off value < 5 mg/L) and erythrocyte sedimentation ratio (ESR) (cut off value <12 mm/h) were assessed simultaneously with other routine laboratory tests. ESR was assessed by Westergren method and CRP level—using the immunoturbidimetric method.

The activity of the disease was established by JADAS-27 (11). Depending on the JIA subtype, the inactive disease was considered with JADAS-27 scores—children with oligoarthritis—≤1, low disease activity—1.1–2, moderate—2.1–4.2 and high—>4.2, and, respectively, for the polyarticular, enthesistis related arthritis (ERA), systemic JIA and other JIA subtype—inactive—≤1, with low disease activity—1.1–3.8, moderate—3.9–8.5 and high—>8.5.

## Statistical analysis

Collected data were analysed with STATISTICA software (Poland). For continuous variables, their distributions were assessed using Shapiro-Wilk test. Due to most continuous characteristics having a non-normal distribution (CRP, ESR) or being measured on an ordinal scale, we proceeded with non-parametric approach. For paired observation, Wilcoxon test was applied and results were reported as before/after medians with 25%–75% ranges and median differences (with 25-75% range). In case of heavily 0-centred distributions where median values were uninformative, the variables were categorized and numbers and percentages were provided additionally.

Categorical data were assessed and presented as numbers and percentages. For paired comparison, McNemmar's test was used. Unpaired comparisons such as frequencies of adverse effects between different treatment regimens were performed using Chi^2^ test.

In all analyses, alfa for declaring statistical significance was chosen as 0.05.

## Results

### Characteristics of the study group

The study included 43 patients aged 5.4 to 17.2 years (Median 14.6 years), 17 (39,5%) males and 26 (60,5%) female already diagnosed with JIA and vaccinated against COVID-19.

The study group was assessed as a whole and divided into two subgroups depending on the age: 5–11 and 12–18 year. The reason for dividing the children into two groups was the fact of different approval time for the COVID-19 vaccine for these age groups. Majority (37/43%–86%) of children from study group was 12–18 years old and only 6/43 (14%) were 5–11 years old. Eight (18.61%) patients had remission without any treatment in the time of the study inclusion. More than a half of the children (24/43–55.81%) has been treated with cDMARDs and 11/43 (25.58%) with bDMARDs and cDMARDs. Fifteen children (15/43) were taking more than one DMARDs and two were on steroids (1—with methotresate, 1—with bDMARD and cyclosporine). Characteristic of group is summarised in (Table 1).

**Table 1:** Characteristics of the study group .

Characteristic	Groups	Total (*N* = 43)	12–18 years (*N* = 37)	5–11 years (*N* = 6)
Continuous variables		Median (25%–75%)		
	Age	14.6 (12.5–17)	15 (13.33–17.0)	8.63 (6.67–10.5)
Categorical variables		*N* (%)		
Gender (*N* = 43)	Male	16 (37.21%)	12 (32.43%)	4 (66.67%)
	Female	27 (72.79%)	25 (67.57%)	2 (33.33%)
JIA subtype (*N* = 43)	Systemic	3 (6.98%)	2 (5.41%)	1 (16.67%)
	Polyarticular	12 (27.91%)	10 (27.03%) (2 female seropositive—RF+)	2 (33,33%)
	Oligoarticular	12 (27.91%)	11 (29.73%)	1 (16.67%)
	ERA	14 (32.56%)	13 (35.14%)	1 (16.67%)
	Other	2 (4.65%)	1 (2.70%)	1 (16.67%)
Treatment	cDMARDs (total)	35	29	6
	*Methotrexate*	25	21	4
	*Sulfasalazine*	11	9	2
	*Others*	3	1	2
	Corticosteroids (GCS)	2	1	1
	bDMARDs (total)	12	11	1
	*Adalimumab*	6	6	0
	*Etanercept*	2	2	0
	*Tocilizumab*	3	2	1
No treatment		8	8	0

JIA, juvenile idiopathic arthritis; ERA, enthesitis related arthritis; cDMARDs, conventional disease-modifying antirheumatic drugs; bDMARDs, biological disease-modifying antirheumatic drugs.

### COVID-19 vaccine adverse events

Vaccine adverse events are classified as local, systemic, or allergic.

**Figure 1:**
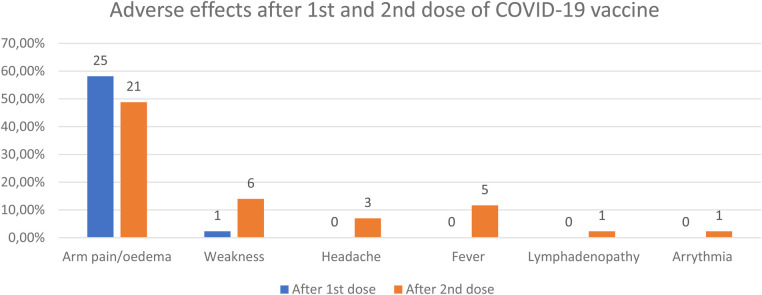
Adverse events after 1st and 2nd dose of COVID-19 vaccine.

After first dose of COVID-19 vaccine 58.14% (25/43) of study group presented mild adverse events: arm pain or oedema at the application side (25/43) and one additionally: weakness. Also 25/43 children (58.14%) had adverse events after second dose of COVID-19 vaccine, however the spectrum of the symptoms was wider. Most of these were local reactions (arm pain or oedema at the application side) in 21/43 patients and mild to moderate systemic adverse events, like: weakness, headache, fever, and in individual patient: lymphadenopathy and arrythmia (it also occurred prior to vaccination) (Tables 2–4, Figure 1). No patients experienced an allergic reaction or a serious adverse event following COVID-19 vaccination. None need to be hospitalized. All symptoms completely resolved.

**Table 2:** Adverse events (AE) after COVID-19 vaccine in JIA patients.

Adverse events (AE)	Types of AE (*N* = 43)	Variable	Total (*N* = 43)	Age 12–18 years (*N* = 37)	Age 5–11 years (*N* = 6)
AE after 1st dose of COVID-19 vaccine	Local	Arm pain or oedema	25 (56.81%)	22 (59.46%)	3 (50%)
	Systemic	Weakness	1 (2.28%)	1 (2.70%)	0 (0%)
	Number of AE	0	18 (41.86%)	15 (40.54%)	3 (50%)
		1	24 (55.81%)	21 (56.76%)	3 (50%)
		2	1 (2.33%)	1 (2.70%)	0 (0%)
AE after 2nd dose of COVID-19 vaccine	Local	Arm pain or oedema	21 (47.73%)	17 (45.95%)	3 (50%)
	Systemic	Weakness	6 (13.95%)	5 (13.51%)	4 (66.67%)
		Headache	3 (6.98%)	3 (8.11%)	0 (0%)
		Fever	5 (11.62%)	4 (10.81%)	1 (16.67%)
		Lymphadenopathy	1 (2.32%)	1 (2.70%)	0 (0%)
		Arrhythmia	1 (2.32%)	0 (0%)	1 (16.67%)
	Number of AE	0	18 (41.86%)	15 (40,54%)	3 (50%)
		1	19 (44.19%)	16 (43.24%)	3 (50%)
		2	1 (2.33%)	1 (2.70%)	0 (0%)
		3	4 (9.30%)	4 (10.81%)	0 (0%)
		4	1 (2.33%)	0 (0%)	1 (16.67%)

**Table 3:** Adverse events after vaccination and JIA treatment.

AE	Total (*N* = 43)	No treatment (*N* = 8)	cDMARDs (*N* = 24)	bDMARDs (*N* = 11)	*p*-value
**After 1st dose**					
Arm pain/oedema	26 (60.47%)	6 (75%)	12 (50%)	8 (72.73%)	0.889
Weakness	1 (2.33%)	1 (12.5%)	0 (0%)	0 (0%)	
**After 2nd dose**					
Arm pain/oedema	21 (48.84%)	4 (50%)	11 (45.83%)	6 (54.54%)	0.432
Weakness	6 (13.95%)	1 (12.5%)	4 (16.67%)	1 (9.09%)	0.820
Lymphadenopathy	1 (2.33%)	0 (0%)	1 (4.17%)	0 (0%)	0.667
Arrythmia	1 (2.33%)	0 (0%)	0 (0%)	1 (9.09%)	0.226
Fever	5 (11.63%)	1 (12.5%)	2 (8.33%)	2 (18.18%)	0.698
headache	3 (6.98%)	1 (12.5%)	2 (8.33%)	0 (0%)	0.374

cDMARDs, conventional disease-modifying antirheumatic drugs; bDMARDs, biological disease-modifying antirheumatic drugs.

**Table 4:** Number of adverse events (AE) after vaccination and JIA treatment DMARD.

AE number	Total (*N* = 43)	No treatment (*N* = 8)	cDMARDs (*N* = 24)	bDMARDs (*N* = 11)	*p*-value
**After 1st dose**					
0	22 (51.16%)	5 (45.45%)	13 (54.17%)	4 (50%)	0.8894
1	20 (46.51%)	6 (54.55%)	11 (45.83%)	3 (37.5%)	
2	1 (2.33%)	0 (%)	0 (%)	1 (12.5%)	
**After 2nd dose**					
0	18 (41.86%)	4 (36.36%)	11 (45.83%)	3 (37.5%)	0.6637
1	19 (44.19%)	6 (54.55%)	9 (37.5%)	4 (50%)	
2	1 (2.33%)	0 (%)	1 (4.17%)	0 (%)	
3	4 (9.30%)	0 (%)	3 (12.5%)	1 (12.5%)	
4	1 (2.33%)	1 (9.09%)	0 (%)	0 (%)	

cDMARDs, conventional disease-modifying antirheumatic drugs; bDMARDs, biological disease-modifying antirheumatic drugs.

### JIA activity before and after COVID-19 vaccination

There were no statistically significant differences in children`s ESR and CRP values before and 6 weeks after COVID-19 vaccination. Likewise, the patient/parent and rheumatologist disease activity assessment according to VAS scale did not change significantly, as well as, there was no change in the number of active joints count determined by the rheumatologist, before and after COVID-19 vaccination.

The majority of children from the study group presented inactive JIA before vaccination (70%). Even more (81.4%) reported with inactive disease 6 weeks after COVID-19 vaccination, however the difference was not statistically significant (*p* = 0.13).

**Figure 2:**
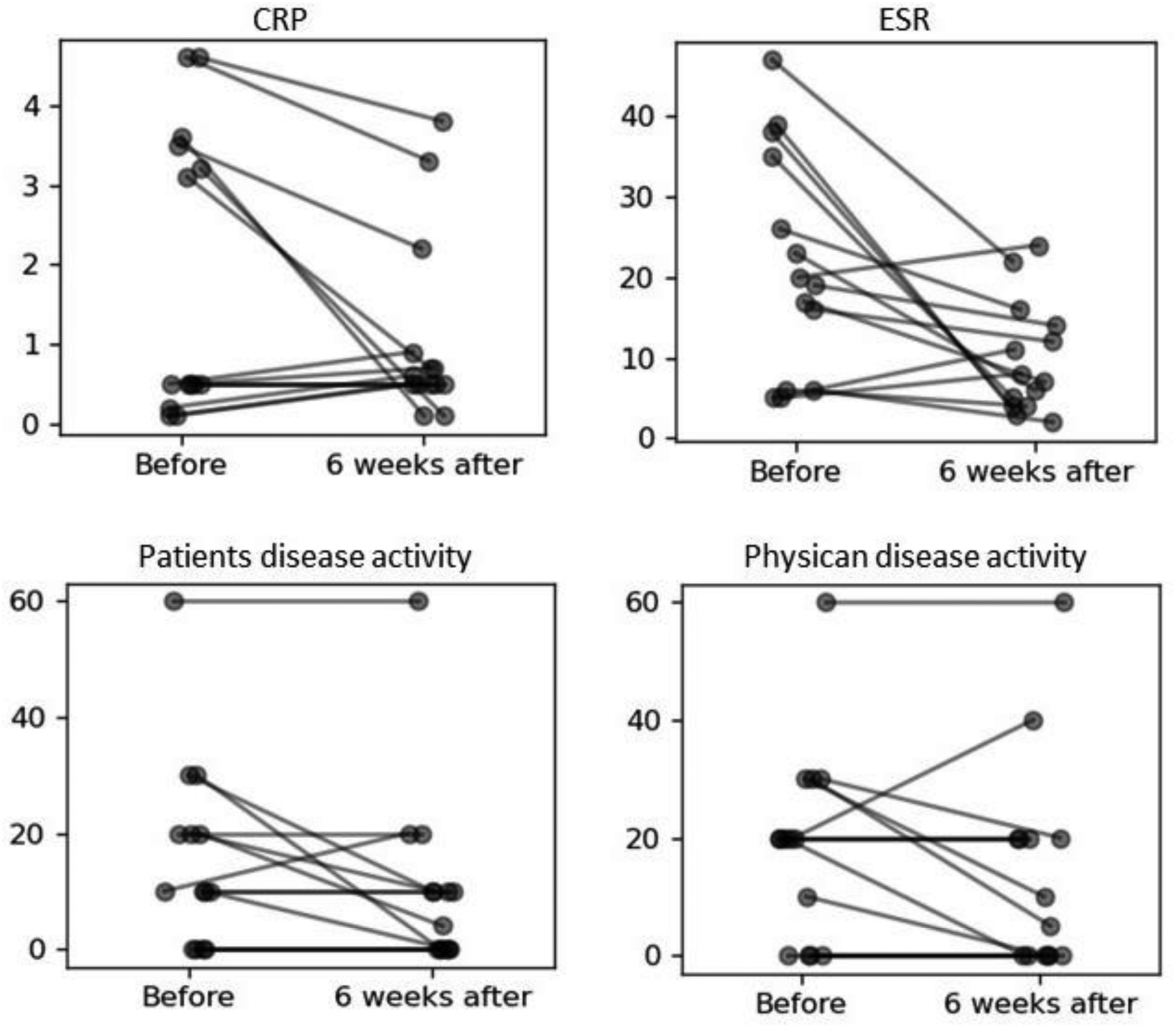
Change of values of CRP, ESR, patients disease activity (PDA) and physician disease activity (PhDA) before and 6 weeks after COVID-19 vaccination.

In the group of patients with JIA with active disease (30.23%) before COVID-19 vaccination, three children (23.1%) had low disease activity, eight (61.5%)—moderate, and two (15.4%)—high according to JADAS-27. Six weeks after COVID-19 vaccination, disease activity decreased significantly (*p* = 0.047), only 8 (18.6%) children presented active disease, with 3 (37.5%) showing low disease activity, 3 (37.5%)—moderate and 2 (25%)—high (Table 5, Figures 1, 2).

**Figure 3:**
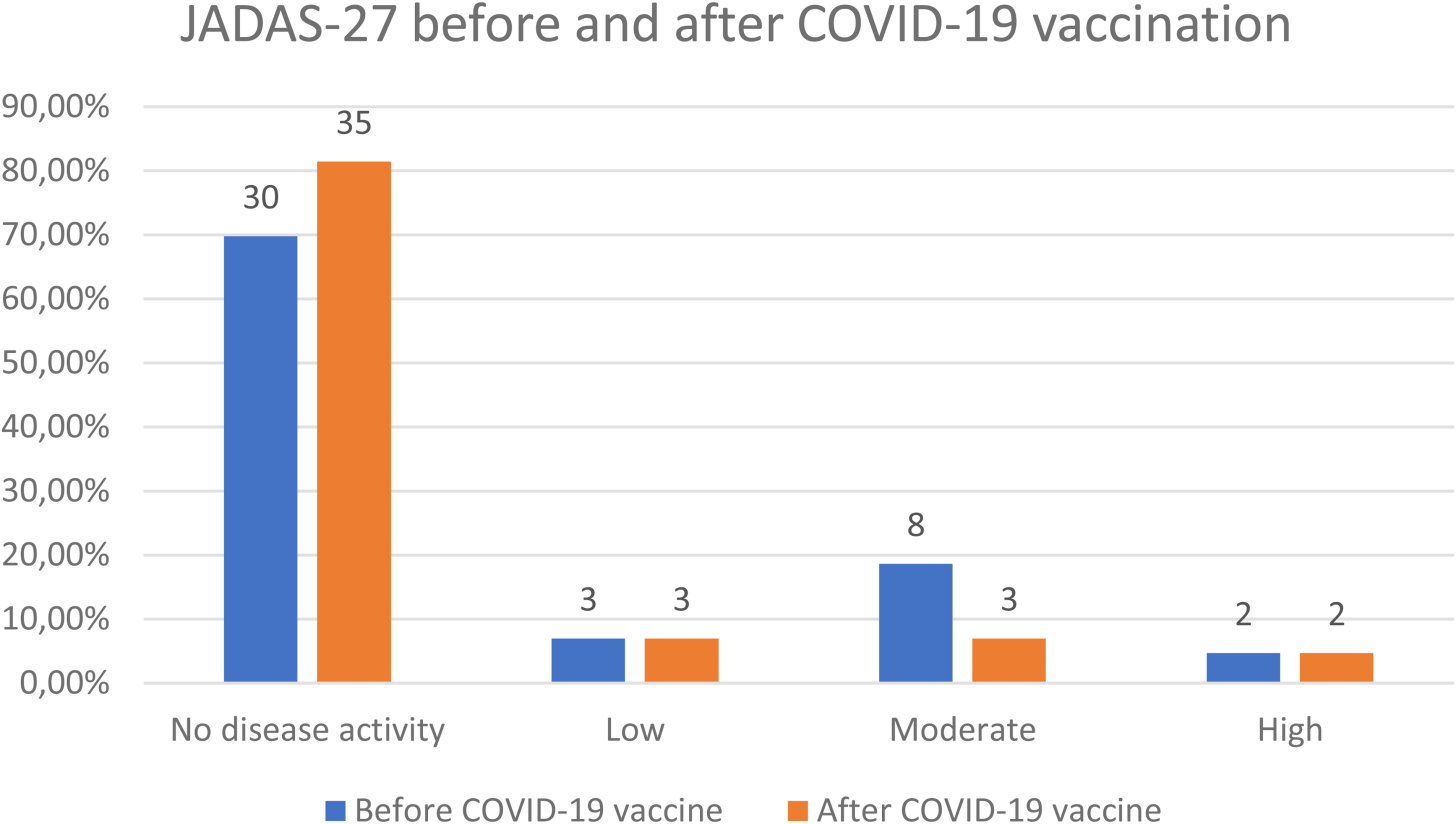
JADAS-27 before and 6 weeks after COVID-19 vaccination.

**Table 5:** JIA activity before and 6 weeks after COVID-19 vaccination.

Variable	Before COVID-19 vaccine	After COVID-19 vaccine	*p*-value
Number of patients with active disease (*N* = 43)	13 (30.23%)	8 (18.60%)	0.130
JADAS-27*			0.047
No disease activity	30 (69.77%)	35 (81.40%)	
Low	3 (9.98%)	3 (6.98%)	
Moderate	8 (18.61%)	3 (6.98%)	
High	2 (4.65%)	2 (4.65%)	

*Disease activity category according to JADAS-27 scale was ranked and compared in a paired scenario using Wilcoxon`s test.

### COVID-19 before and after vaccination

Ten out of 43 (22.72%) children with JIA had a known history of COVID-19 infection prior to COVID-19 vaccination. All of these patients were symptomatic and presented mild symptoms. During the 3-month follow-up period after vaccination, none of the patients developed COVID-19.

### Mandatory and recommended vaccinations

All the patients also previously received all mandatory vaccinations (according to the Polish immunization program). What is more 15 (34.88%) patients were also vaccinated with one or more additional recommended vaccinations. None of the study group patients reported the occurrence of an undesirable adverse event after a previous mandatory or recommended vaccinations.

### COVID-19 vaccination in parents and siblings

All the parents and the all eligible siblings (33, 100%) at the appropriate age have been vaccinated for COVID-19. None of the parents and siblings of our patients experienced an allergic reaction or a serious adverse events following COVID-19 vaccination.

## Discussion

Our study demonstrated a good vaccine safety profile in JIA patients. None of the patients experienced an allergic reaction or other form of serious adverse events following COVID-19 vaccination. After the first dose of COVID-19 vaccine more than a half study group presented mild adverse events: arm pain or oedema at the application side, one patient reported additionally weakness. Also more than half of the children (58.14%) had adverse events after second dose of COVID-19 vaccine, however the spectrum of the symptoms was wider. Most of these were local reactions (arm pain or oedema at the application side) and mild to moderate systemic adverse events: weakness, headache, fever, in one patient lymphadenopathy and in one—arrythmia. Myocarditis and pericarditis have rarely been reported and when reported, the cases have especially been in adolescents and young adult males within several days after mRNA COVID-19 vaccination (Pfizer-BioNTech or Moderna)—usually within a week of vaccination and more often after the second dose (12). All symptoms completely resolved. The incidence of adverse events following COVID-19 vaccination was not dependent on the treatment used in patients.

Our observation did not show a negative effect of COVID-19 vaccination on disease activity. We even saw a slight improvement comparing to the pre-vaccination assessment, that was probably connected with longer period of JIA treatment, however indicated that vaccination did not exacerbate disease course. During the 3-month follow-up period after vaccination, none of the patients developed SARS-CoV-2 infection.

We are aware that not all children with rheumatic diseases have been vaccinated despite the recommendation. Nevertheless, we would like to point out that all parents and all eligible siblings of included patients with JIA had been vaccinated against COVID-19. None of them experienced an allergic reaction or a serious adverse events following COVID-19 vaccination.

In May 2021, the first COVID-19 vaccine (Pfizer-BioNTech), was approved by the European Medicines Agency (EMA) for children aged 12 years and older, followed by another vaccine, (Moderna vaccine), in July 2021. In November 30, 2021 EMA approved Pfizer-BioNTech vaccine for use also in children 5 years of age and older in Europe (13, 14).

In Poland, vaccination against COVID-19 for adults and adolescents over 16 years of age began on December 27, 2020, as in other European Union countries. According to the Republic of Poland website gov.pl (15), more than 54 million vaccines were administered until 4 July 2022. Currently, 22,516,851 people are fully vaccinated, representing 59.9% of total Polish population. On June 7, 2021, the 1st COVID-19 vaccine for children aged 12–15 years was approved in Poland, and on December 12, 2021 also for children from the age of 5. On January 22, 2022, a booster dose was approved for adolescents 12–15 years of age. By July 4, 2022, approximately 3,3 million doses were administered in the 5–17 age group. According to Statistics Poland (16), the population aged 0–17 is 6.9 million, which means that many children and adolescents remain unvaccinated.

Both, PReS and Polish Society of Rheumatology recommended vaccinations for children with rheumatic diseases (17).

There are few reports on the safety and efficacy of vaccination against COVID-19 in children and adolescents with rheumatic diseases. Physician and guardians are concerned about the safety of patients, both in terms of adverse events and the impact on the activity of the disease.

Haslak, et al. indicated in similar like our study an acceptable safety profile of mRNA COVID-19 vaccines in children with inflammatory rheumatic diseases. They monitored 246 patients (receiving biological or other therapies) aged 12—20.1 years. The most common adverse events were fatigue (27.6%), headache (17.9%), myalgia (15.4%), arthralgia (15.4%) and fever (14.2%). Only 3 subjects (2 patients with familial mediterranean fever, and one healthy child) were considered to have experienced serious adverse events requiring hospitalization. Local reactions were seen in 8.1%, and 12.1% children had disease flares within 1 month after the vaccines. There was no significant relationship between adverse events frequency and age, gender, activity of the diseases and treatment (18).

Dimopoulou, et al. evaluated the immunogenicity of the COVID-19 vaccine in 21 adolescents with JIA aged 16–21years on TNF-inhibitors treatment (15—together with methotrexate). All patients developed a sustained humoral response against SARS-CoV-2 assessed 1 and 3 months after vaccination. None of the participants developed disease flare during the 3 months follow-up period (19) similar to our observations. Local reactions were more frequent (74%) that in ours group, but systemic reactions were relatively infrequent (19%). As in our study, the most of localized and systemic reactions were noted after the second dose of the vaccine. Also, no exacerbation of underlying disease was noted, based on evaluation of the JADAS-27 scale at 1 month before the vaccination, as well as at 1 and 3 months after the second dose (20).

Heshin-Bekenstein, et al. presented their observations on the safety and immunogenicity of the anti-SARS-CoV-2 vaccine in 91 patients: adolescents (ages 12–18 years) and young adults ages 18–21 years) with juvenile-onset inflammatory rheumatic diseases, including 42 with JIA, and 80% of those patients were receiving immunomodulating drugs. Authors reported good safety profile: 96.7% of patients reported mild or no side-effects, and no change in disease activity. However, 3 patients had transient acute symptoms: renal failure and pulmonary haemorrhage following the first vaccination and mild lupus flare and viral infection following the second dose. Likewise in our study no cases of COVID-19 were documented during the 3-month follow-up (21).

Also, the observations of Arslanoglu, et al. demonstrated a good safety profile of the COVID-19 vaccine in 228 children with rheumatic diseases (99 patients with JIA), regardless the treatment (conventional or biological). Adverse events after the first dose of vaccine were recorded in 93 (54.7%) patients and in 66 (52.4%)—after the second dose. No serious adverse events including myocarditis, anaphylactic shock, or death were observed (22). This study also confirmed our observations that vaccines should not cause flares of rheumatic diseases in children and adolescents.

There are some limitations of the study. There was no control group of healthy children included into the study. Moreover, the observational period was short—maximum 3-month follow-up. Part of the children got only one dose of vaccine, mainly those who were younger than 12 years old, as the COVID-19 vaccine for the children 5–11 years was available later than for those over 12 years. It would be valuable to provide data of serological profile of patients, in order to demonstrate the efficacy of vaccine, however it was the questionnaire study.

Summing up, our study and all the studies published so far confirm the safety of COVID-19 vaccine in pediatric population. Our group of patients covers children with different age than previously published. Nevertheless, although the published studies differ in the age of the observed patients, the type of diagnoses and applied therapies, all of them, together with ours, indicate the safety of COVID-19 vaccines in children and adolescents with rheumatic diseases. We are aware that not all of patients have been vaccinated despite the recommendation due to still concerns among parents about COVID-19 vaccine safety. We hope our study will help alleviate the doubts of patients, parents and some healthcare professionals about this important vaccination. Especially that we have reasons to be afraid of the next wave of the SARS-CoV-2 infections.

## Conclusions

Our study found that children and adolescents with JIA with remission without treatment or on the long-term treatment—cDMARDs or even bDMARDs, can be safely vaccinated for COVID-19. Moreover, we found no evidence that COVID-19 vaccination does interfere with the JIA treatment and can exacerbate symptoms of the disease. Vaccination protected against developing COVID-19 during follow-up.

The presented analysis of our local Polish experience may become helpful in everyday practice of pediatric rheumatologist who may refer to the findings of this study during conversation with patients or parents regarding the efficacy and safety of COVID-19 vaccines. Our observations are also important for future vaccination recommendations in JIA patients.

## Data Availability

The raw data supporting the conclusions of this article will be made available by the authors, without undue reservation.
